# An experimental study to investigate typical temperature conditions in fuel tanks of European vehicles

**DOI:** 10.1007/s11356-019-04985-7

**Published:** 2019-04-25

**Authors:** Theodoros Grigoratos, Giorgio Martini, Massimo Carriero

**Affiliations:** Joint Research Centre (JRC), Directorate for Energy, Transport and Climate (IETC), Sustainable Transport Unit (STU), European Commission, Via E Fermi 2749, 21027 Ispra, Italy

**Keywords:** Evaporative emissions, Running losses, Fuel tank temperature, Purging flow rate

## Abstract

Vehicular evaporative emissions have been recognized as an important source of volatile organic compounds to the environment and are of high environmental concern since these compounds have been associated to the formation of surface ozone and secondary organic aerosols. Evaporative emissions occur during any vehicle operation. In Europe, a revised legislative test procedure has been recently introduced to better control evaporative emissions during parking. However, emissions related to normal driving conditions—the so-called running losses—have received less attention compared with the other categories. The current study aims at giving some insights to the prevailing temperature conditions in fuel tanks of typical European vehicles during normal driving operation. The effects of ambient air temperature, trip duration, vehicle speed, and fuel tank level on the temperature reached by the fuel inside the tank under different real-world operating conditions were studied. Tank temperature can exceed 40 °C depending on ambient and driving conditions. Ambient temperature was found to be the most important parameter affecting the tank temperature. Trip duration and driving pattern may also have an influence on the tank temperature particularly when long trips combined with high vehicle speed are examined. Additionally, the difference between tank and ambient temperature was examined during the individual trips and was found to vary between 1 and 10 °C depending on the testing conditions. The most important parameters affecting the delta temperature were found to be the trip duration and the maximum vehicle speed. Finally, the purging strategy of two of the test vehicles was monitored, and the parameters affecting the purging flow rate were investigated. No strong correlation between the canister flow rate with ambient temperature, vehicle speed, or fuel level was observed in either of the tested vehicles. Substantially different canister flow rate levels between the two vehicles point to different purging strategies.

**Electronic supplementary material:**

The online version of this article (10.1007/s11356-019-04985-7) contains supplementary material, which is available to authorized users.

## Introduction

Evaporative emissions from vehicles consist of volatile organic compound (VOC) emissions not linked to the combustion process of the fuel inside thermal engines. US EPA has provided a list of compounds considered relevant for evaporative emissions (EPA 2014), while the most abundant VOC species have been described in detail elsewhere (Yue et al. 2017). Evaporative emissions are a concern mainly for petrol vehicles due to the low boiling point of the fuel while they are negligible for diesel vehicles due to the very low vapor pressure of the diesel fuel. In the USA, as well as some developed East Asian countries, the vast majority of the passenger vehicles are composed of gasoline engine; therefore, the topic of evaporative emissions is completely relevant. In Europe, the European Automobile Manufacturers Association (ACEA) recently announced a 5-year tendency for increase of the petrol vehicle share with 49.4% of total passenger car registrations in 2017 (EU-15) being composed of gasoline engine (ACEA 2018). Practically, one out of two newly registered passenger cars is a petrol vehicle highlighting the need for considering evaporative emissions. In petrol vehicles, the most important potential source of evaporative emissions is the loss of fuel from the fuel system through evaporation and permeation. US EPA categorizes evaporative emissions based on the evaporative mechanism with the following processes: permeation (the migration of hydrocarbons through materials in the fuel system), tank vapor venting (TVV—vapor generated in fuel system lost to the atmosphere when not contained by evaporative emissions control system), liquid leaks (liquid fuel leaking from the fuel system ultimately evaporating), and refueling emissions (spillage and vapor displacement as a result of refueling). Other sources of VOCs are the so-called background emissions and are direct emissions from the vehicles’ paint, tires, plastic components, interior trims, or other fluids and tend to be very small compared with evaporation and permeation emissions (Hata et al. 2018).

Evaporative emissions are of high environmental concern since VOCs have been proven to play a key role in the formation of surface ozone (Seinfeld and Pandis 2016) and secondary organic aerosols (SOAs) (Kroll and Seinfeld 2008). Recently, more and more studies have been evaluating evaporative emissions from modern vehicles. For instance, Martini et al. (2014) calculated annual evaporative emissions from typical European passenger cars at approximately 1000 g/vehicle. Similarly, Yamada et al. (2015) estimated annual evaporative emissions of approximately 450 g/vehicle in Japan, while Liu et al. (2015) reported an average value of about 500 g/vehicle for Chinese passenger cars. Zhu et al. (2017) reported that evaporative emissions from China IV vehicles were 1.1–1.4 times higher than the tailpipe HC emissions, whereas their annual evaporative emissions were almost 1.8–2.8 times higher than those from the tier 2 vehicle. Yue et al. (2017) provides a summary of all related studies highlighting the importance of evaporative emissions worldwide.

Fuel-related evaporative emissions may occur during any vehicle operation including parking (diurnal breathing loss (DBL)), normal driving (running loss (RL)), vehicle being stopped after running operation (hot soak loss (HSL)), and vehicle refueling (Yamada 2013). The two main mechanisms causing evaporative emissions during vehicle use are evaporation and permeation. Permeation does not occur through a specific opening; instead, individual fuel molecules penetrate (i.e., they effectively mix with) the walls of plastic and rubber components like hoses, seals, and non-metallic fuel tanks and eventually find their way to the outside. They differ from leaks in that they occur on the molecular level and do not represent a failure of any kind in a specific location. Fuel permeation is highly relevant mainly for plastic or elastomeric materials, depends strongly from the temperature, and usually occurs in any vehicle operating conditions (EPA 2014).

Fuel evaporation is linked to the temperature reached by the fuel (*T*_tank_) and can occur both during parking events or driving operation. Ambient air and road surface temperature as well as solar radiation represent the main heat sources that may lead to a significant increase of the *T*_tank_ during parking. The fuel tank—by design—is usually vented to the atmosphere through a pressure relief valve so that the tank pressure is maintained slightly above atmospheric. If the pressure inside the tank rises above that value, a mixture of air and petrol vapors may be released into the air. In modern vehicles, the tank is vented through an activated carbon canister that adsorbs and stores the hydrocarbons preventing emissions to the atmosphere. More than 95% of fuel vapor generation is reported to be trapped by the carbon canister; however, it has a limited adsorbing capacity and has to be periodically purged to desorb the stored hydrocarbons (Yamada 2013). On the other hand, during driving operation, ambient air and road surface temperature, solar radiation, hot engine and exhaust system, fuel pump and fuel return—if present—represent the main heat sources that may lead to a significant increase of the *T*_tank_, and therefore to the evaporation of the lightest petrol fractions with a corresponding increase of the pressure inside the tank. US EPA defines running loss emissions as evaporative hydrocarbons that are emitted when the vehicle is in operation (EPA 1999). In other words, running loss emissions consist of vapor venting during vehicle operation (EPA 2014). The main mechanism for running losses in modern vehicles includes the emission of small amounts of HCs from the fuel cap and vapor canister due to the increase of the *T*_tank_. Most of these HCs are captured by the canister. However, during driving operation, part of the combustion air flows through the canister removing these hydrocarbons and routing them back through the intake of the engine to be consumed during combustion. In specific cases, the balance among the fuel evaporation rate, the amount of fuel being pumped to the engine, and the purge flow rate through the canister are such that there is a net flow of air/fuel vapors escaping the tank through the vent and the canister. If the canister is already saturated, the fuel vapors will be released into the air. These emissions occur only during driving and are known as running losses. Another important source of evaporative emissions is the refueling operation. When liquid fuel is delivered into the tank, the air/petrol vapor mixture present in the tank is displaced and may be released into the air. Refueling emissions are described in details elsewhere (Yamada et al. 2018) and are out of the scope of the current study.

The different sources of evaporative emissions are regulated and therefore controlled in different ways around the world. In the USA, all different sources of emissions are addressed by specific test procedures. Hot soak and diurnal tests carried out in Sealed House for Evaporative Determination (SHED) address evaporative emissions during parking events. Running losses and refueling emissions are also checked by means of specific tests. Details regarding the methods can be found elsewhere (EPA 2014). China’s new emission standard reduces evaporative emissions from 2 to 0.7 g/day and will become effective in 2020. It includes a pre-conditioning for hot soak testing at high temperature (38 ± 2 °C) following WLTC cycle and a 12–36-h high-temperature soak (38 ± 2 °C) before a high-temperature driving test (Man et al. 2018). In Europe, evaporative emissions during parking events are measured with a recently modified test procedure which is quite similar to the US 2-day-long diurnal test and has introduced specific provisions to reduce fuel permeation. The new European legislative test procedure to determine evaporative emissions is based on the so-called diurnal test (Regulation 2017/1221). The method is described in detail elsewhere (EC 2017). On the other hand, refueling emissions are controlled by means of the so-called stage II vapor recovery system. The fuel nozzle is designed to draw the air/petrol vapor mixture displaced by the liquid fuel entering the tank and to route it to the underground petrol storage tank of the service station. Finally, running losses are not currently regulated in Europe. The main reason is that so far the average European temperature conditions have been considered not to be critical for the European cars in order to lead to excessive pressure values inside the tank during trips.

The objective of this study is to investigate the temperatures that can be reached by the fuel inside the tank for different vehicles tested in real world under different operating conditions. Parameters like ambient air temperature, trip duration, vehicle speed, and fuel tank level are investigated with the aim of understanding their influence on the *T*_tank_ for different vehicles. This investigation aims in providing some insights for better understanding evaporative emissions and more particularly running losses of modern petrol vehicles. For that reason also, the purging strategy for two of the test vehicles was monitored and some preliminary conclusions were reached.

## Experimental setup

Real-world tank temperatures were measured for seven different vehicles under a wide range of operating conditions. Detailed description of the vehicles, test conditions, and measurement protocol are provided in this chapter.

### Tested vehicles

Seven petrol passenger cars were employed for the purpose of the present study. Studied vehicles cover a wide range of engine specifications including engine displacement of approximately 1000–1600 cm^3^ and horsepower of about 45–110 kW. Some of the vehicles were relatively old (vehicles #1, #4, and #5) since they were manufactured over a decade ago (2004–2007) and fall in the Euro 4–5 classification, while most of them (vehicles #2, #3, #6, and #7) were recently registered (2015–2017) and fall in the Euro 6 classification. One hybrid passenger car was also tested (vehicle #6) in both conventional and hybrid modes. These vehicles can be considered typical of the European fleet in their category. Some of the vehicles are rentals, and they were delivered to JRC without any prior modifications to their original setup, while others were provided by individuals and were tested by them over their daily routine conditions. Table 1 provides some generalized information regarding the specifications of the tested vehicles as well as information regarding the equipment used for testing.

**Table 1:** Main specifications of the vehicles and equipment used for testing

Vehicle (vehicle no.)	Vehicle specifications (cc-kW-year)	Fuel tank capacity (L)	Measurement equipment
Mini Cooper 1 Vehicle #1	1598-66-2004	50	OBD and *T*_tank_ logger
Lancia Y Vehicle #2	1242-51-2015	40	OBD and *T*_tank_ logger
Opel Astra Vehicle #3	999-77-2017	48	OBD and *T*_tank_ logger
Mini Cooper 2 Vehicle #4	1598-85-2005	50	OBD and *T*_tank_ logger
FIAT Panda Vehicle #5	1242-44-2007	35	OBD and *T*_tank_ logger
Golf Hybrid Vehicle #6	1395-110-2015	40	OBD and *T*_tank_ logger and mass flow meter
FORD Fiesta Vehicle #7	998-59-2015	47	OBD and *T*_tank_ logger and mass flow meter

### Instrumentation

Tank temperature was measured in all tested vehicles by means of a high-temperature data logger with a flexible probe (OMEGA/OM-CP-HITEMP140-FP-TSK). The data logger features a flexible RTD probe of 91.4-cm length with a narrow diameter which is installed in the vehicle’s fuel tank. It can measure temperatures up to 140 °C with an accuracy of ± 0.1 °C. The device records and stores up to 32,700 time stamp readings. Data can be viewed in graphical or tabular formats, and summary and statistic views are available for further analysis.

Information regarding the on-board diagnostics of all vehicles was recorded by means of a TEXA OBD Log. The device is connected directly to the vehicle’s diagnostic socket without impeding normal vehicle use and stores information regarding the trips with a sampling resolution of 1 to 5 s. At the end of the trips, recorded data are downloaded using the OBD Log Software Suite. The software allows for viewing and processing of the recorded parameters (i.e., vehicle speed, air temperature, fuel tank level, etc.) after they have been downloaded.

A TSI thermal mass flow meter (TSI Series 4040) was employed for the measurement of the vapor flow in and out of the canister in vehicles #6 and #7. The flow meter measures flow rate in the range of 0–300 L/min and comes with an uncertainty specified as ± 2% of full scale. The flow meter is unidirectional meaning that it does not provide information whether gas vapors are flowing outside the tank or ambient air is sucked in it. The flow meter was connected to an external computer, and data were recorded directly to it.

### Categorization of trips

Real-world tests were performed with the aim of recording the *T*_tank_ of different vehicles under various driving and environmental conditions. More specifically, the influence of trip duration, vehicle average, and maximum speed as well as of *T*_amb_ over the *T*_tank_ was examined. Four of the vehicles (#1, #2, #4, #5) were driven by its owners over their daily routine routes in the general Varese area (Italy). Vehicle #3 was tested only over a motorway route during a trip from Milan to Rome. Vehicles #6 and #7 were employed for the purposes of the study and therefore include more targeted tests under different speed and duration conditions.

In order to categorize the trips based on their duration, vehicle speed, and *T*_amb_, some assumptions were made. Since most of the trips do not include very distinct urban and rural parts, it was decided to distinguish the different trips based on the average vehicle speed. Average trip speeds lower than 30 km/h were attributed to low speed, between 30 and 70 km/h to medium speed, and higher than 70 km/h to high speed. This categorization fits the analysis performed over the worldwide WLTP database for the definition of typical driving conditions (PMP 2016). It should be noted that the attribution of a trip in one speed category does not necessarily mean that the vehicle was not driven under different conditions during this particular trip. It only means that the average velocity over the trip falls in the certain category. For instance, there are cases where the average vehicle speed is calculated to be between 30 and 70 km/h; however, distinct motorway parts are driven during the trip. At the next stage, the trips were also categorized based on their duration to short, medium, and long. Since a standard methodology to categorize trips based on their duration does not exist, it was decided to categorize trips with total duration shorter than 600 s (10 min) as short, trips lasting 600–1800 s as medium, and trips longer than 1800 s (30 min) as long. This categorization does allow to draw some conclusions regarding the influence of the trip duration to the *T*_tank_ particularly when short and long trips are compared with each other.

Environmental conditions were variable during testing. All tests were conducted under dry conditions (without rain events); however, *T*_amb_ varied between different vehicles and in most cases between different trips of the same vehicle. Trips were categorized to low and high temperature based on the average *T*_amb_ recorded during testing. Category low refers to trips conducted with *T*_amb_ under 25 °C, while category high applies to trips conducted at 25 °C or higher. Since all tests were performed in the summer period in most cases, the difference between the two classes reflects the time of the day at which the tests were done. For instance, for vehicles #1, #2, and #5, low-temperature trips mostly refer to early morning tests with *T*_amb_ close to 20 °C, while high category trips refer to noon–afternoon tests with *T*_amb_ close to 30 °C.

Table 2 provides an overview of the categorization of trips for the different vehicles. Apart from the number of trips attributed in each category, also the average value of each parameter in each category is provided in parenthesis (where applicable). For instance, vehicle #5 performed in total ten trips all of which were of medium duration with an average value of 1253 s. The same vehicle did not perform any long trip, and that is why the average long duration value is non-applicable (n/a).

**Table 2:** Categorization of the trips based on their duration, vehicle speed, and *T*_amb_

Vehicle number (total trips)	Total trip duration number of trips (mean duration (s))			Average trip speed number of trips (mean speed (km/h))			Average *T*_amb_ number of trips (mean *T*_amb_ (°C))	
	Short	Medium	Long	Low	Medium	High	Low	High
Vehicle #1 (12)	8 (514)	3 (1179)	1 (1859)	2 (27.8)	10 (36.5)	0 (n/a)	8 (20.7)	4 (27.1)
Vehicle #2 (22)	5 (312)	17 (880)	0 (n/a)	8 (21.4)	14 (37.8)	0 (n/a)	7 (21.5)	15 (30.1)
Vehicle #3 (5)	0 (n/a)	0 (n/a)	5 (5966)	0 (n/a)	1 (58.8)	4 (96.1)	0 (n/a)	5 (29.4)
Vehicle #4 (6)	1 (240)	5 (1167)	0 (n/a)	2 (26.0)	4 (45.5)	0 (n/a)	0 (n/a)	6 (29.5)
Vehicle #5 (10)	0 (n/a)	10 (1253)	0 (n/a)	0 (n/a)	10 (38.9)	0 (n/a)	5 (23.0)	5 (29.4)
Vehicle #6 (6)	0 (n/a)	1 (1127)	5 (4194)	0 (n/a)	4 (41.4)	2 (87.6)	2 (20.5)	4 (27.8)
Vehicle #7 (17)	4 (574)	9 (919)	4 (3200)	2 (21.1)	15 (41.1)	0 (n/a)	5 (23.7)	12 (29.1)

## Results and discussion

### Trip description

Paragraph 2.3 provides details regarding the categorization of the trips while this paragraph tries to summarize the main characteristics of the individual trips for all vehicles. This is considered necessary in order to better analyze and understand the results described in the following chapters.

Vehicles #1, #2, #4, and #5 were driven by their owners over their daily routine routes. As a result, not all possible trip categories were covered since tests were conducted randomly. More specifically, vehicle #1 performed 12 trips over urban and rural conditions most of which were of short duration (< 10 min). All trips were similar to each other and were performed with an average speed of approximately 30 km/h. Most of the trips were done early in the morning or late at evening under generally low *T*_amb_. Some trips were conducted at moderate *T*_amb_ but never higher than 30 °C. Therefore, vehicle #1 was not tested under extreme environmental conditions but also was not tested under very different speed and trip duration conditions. Vehicle #2 performed 22 urban and rural trips mostly of short–medium duration (< 15 min). Some of the trips took place in the morning with relatively low *T*_amb_ (~ 20 °C), while others took place in the noon–afternoon under higher *T*_amb_ (~ 30 °C). High *T*_amb_ trips of vehicle #2 are of particular interest as some of them were performed at temperatures reaching 35 °C. Vehicle #4 was tested over urban and rural conditions usually performing medium duration trips (~ 20 min). Half of the total of six trips were done under moderate *T*_amb_ (~ 26 °C) and the other half over high *T*_amb_ (~ 33 °C) allowing thus for a comparison between different temperature blocks. Vehicle #5 performed ten medium duration trips (~ 20 min) over rural conditions. Trips for this vehicle took place either in the morning or in the afternoon. The first set of morning trips were done under low *T*_amb_ (22–25 °C), while the second set reflect afternoon trips with higher *T*_amb_. However, like in the case of vehicle #1, it was not tested under extreme environmental conditions or under different speed and trip duration conditions. Overall, these vehicles may allow for evaluations regarding the influence of *T*_amb_ to the *T*_tank_ but not for evaluation of the trip duration and vehicle speed effect to the *T*_tank_. The reason is that these vehicles were tested mostly under mixed urban and rural driving conditions, and the tests were of similar duration, thus not allowing for differentiation based on these parameters.

On the other hand, vehicles #3, #6, and #7 were driven solely with the aim of testing them at different speed and trip duration conditions. Indeed, vehicle #3 performed five motorway trips, all of them longer than 30 min. High average speeds were recorded in most of the trips. Two of the trips took place under lower *T*_amb_ of ~ 25 °C, while the rest took place over higher *T*_amb_ reaching 36 °C. Vehicle #6 performed six trips over rural and motorway conditions most of which were of long duration (> 1 h) and relatively high speed. Two of the trips took place in the morning with *T*_amb_ of ~ 20 °C, while the rest were done over higher *T*_amb_ (26–30 °C). Unfortunately, testing days of vehicle #6 were not very hot in order to investigate the combined effect of long duration, high vehicle speed, and high *T*_amb_ to the *T*_tank_. Finally, vehicle #7 performed 17 medium speed trips of different duration. Some of the trips were conducted in the morning with relatively lower *T*_amb_ (~ 24 °C), while others took place at noon–afternoon under higher *T*_amb_ (~ 29 °C). Overall, a wide range of speed and duration as well as *T*_amb_ conditions was tested with vehicles #3, #6, and #7.

**Fig. 1:**
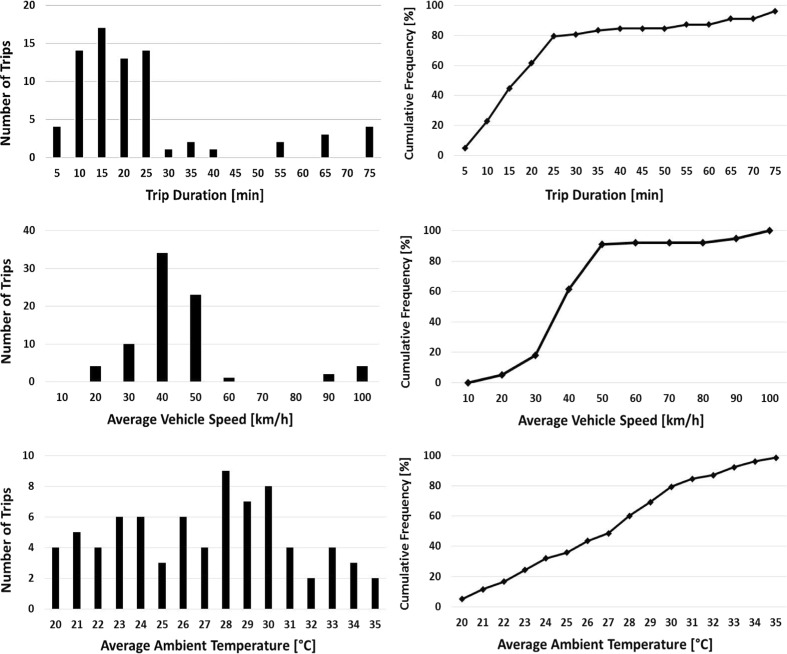
Frequency and cumulative distributions of trip duration, average vehicle speed, and average ambient temperature for all tested vehicles

Figure 1 depicts the frequency distribution as well as the cumulative distributions for trip duration, average vehicle speed, and average ambient temperature cumulated for all tested vehicles. Approximately 25% of the 78 trips are of low duration (< 10 min), while about 20% are longer than 30 min. Similarly, approximately 20% of the trips take place under low average speed (< 30 km/h), while less than 10% are being conducted over high average speed (> 70 km/h). Finally, there is a wide distribution of *T*_amb_ during testing with the average testing temperature being at 27 °C and 10% of the trips being conducted at average *T*_amb_ higher than 33 °C.

### Tank temperature vs. ambient temperature profile

**Fig. 2:**
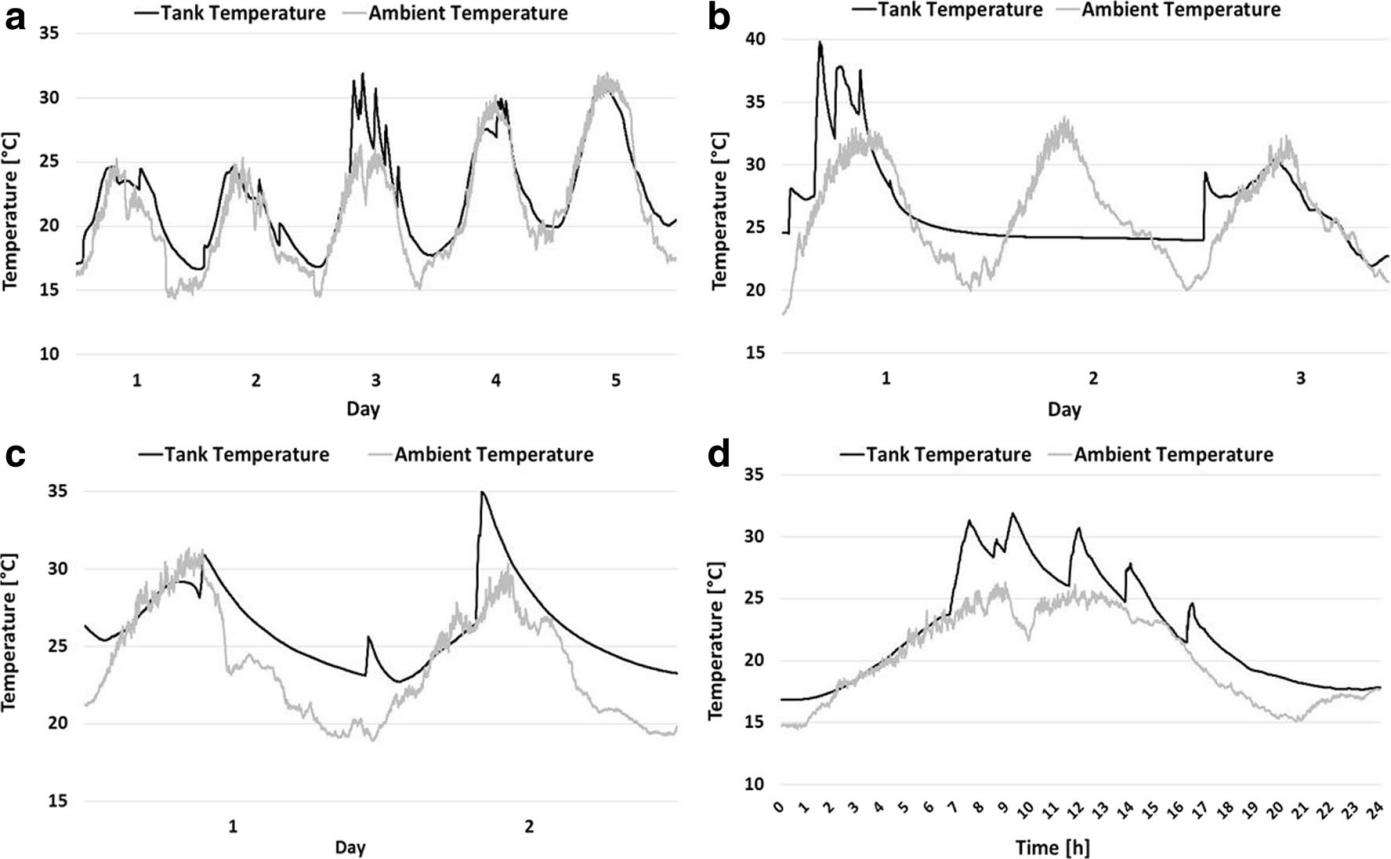
Continuous *T*_amb_ and *T*_tank_ for vehicles: **a** #1 for five consecutive days (driving and parked outside), **b** #7 for three consecutive days (driving and parked inside), **c** #2 for two consecutive days (driving and parked outside), and **d** #1 for 1 day (driving and parked outside)

Figure 2 shows some examples of continuous *T*_amb_ and *T*_tank_ recordings over consecutive days for different vehicles. Twenty-four-hour *T*_amb_ data were retrieved from the official JRC metro station, while *T*_tank_ was anyway monitored at a 24-h basis with a sampling frequency of 1 min. Since some of the vehicles were traveling in the general Varese area and there could be deviations to the actual *T*_amb_ with respect to that recorded at the JRC station, only vehicles moving in the JRC area are considered in Fig. 2.

Figure 2a depicts 5 days of continuous *T*_amb_ and *T*_tank_ recordings for vehicle #1. Recordings with this particular vehicle were taken during driving and when the vehicle was parked in an open space. It is demonstrated that *T*_tank_ follows the trend of *T*_amb_ with some deviations occurring during driving. In other words, when the vehicle moves or is parked in an open space, *T*_tank_ follows the trend of *T*_amb_. This was confirmed for all trips and all tested vehicles for which continuous *T*_amb_ data is available and is supported by previous literature data (Yamada 2013). This is very important since generally high *T*_amb_ (> 35 °C) like the ones recorded in Southern Europe during summer would result in equally high or even higher *T*_tank_, and therefore potential release of evaporative emissions to the environment particularly when the vehicles are being parked outside for many consecutive days (EPA 2014).

The picture changes when a vehicle is parked indoors. Figure 2b shows *T*_amb_ and *T*_tank_ recordings of three consecutive days for vehicle #7. In this case, recordings were taken during driving as well as when the vehicle was parked indoors. This is why *T*_tank_ in the beginning of day 1—and before testing—is much higher than the *T*_amb_ (25 °C vs. 18 °C). Actually, the temperature of the parking space is quite stable at 24 °C during the summer period; therefore, it is not expected to influence the *T*_tank_. Indeed, *T*_tank_ does not change at all during the entire second day (~ 24 °C). Overall, it is concluded that *T*_tank_ of vehicles parked indoors does not follow the *T*_amb_ but is stabilized to a temperature reflecting that of the closed space.

*T*_tank_ differentiates from *T*_amb_ not only when the vehicle is parked indoors but also during running operation and particularly when multiple consecutive trips take place. Figure 2c depicts a continuous 2-day recording of *T*_amb_ and *T*_tank_ for vehicle #2 and shows that *T*_tank_ differentiates significantly from *T*_amb_ during testing (day 2). Recordings with vehicle #2 were taken during driving and parking in an open space. The same effect is also observed to vehicles #1 (Fig. 2a) and #7 (Fig. 2b) during days 3 and 1, respectively, as well as to all tested vehicles when actual trips are conducted. The increase of *T*_tank_ can vary between 1 and 10 °C and depends upon trip duration, vehicle speed, and *T*_amb_. More data regarding the influence of these parameters to the *T*_tank_ will be discussed to the next chapter.

Regarding multiple consecutive trips, Figure 2d depicts 1-day continuous data of vehicle #1 (driving and parking outside) and focuses on the *T*_tank_ behavior during multiple consecutive trips. It is seen that in some cases, *T*_tank_ does not have enough time to decrease and reach the actual *T*_amb_ after a trip (HSL). As a consequence, the next trip starts with the *T*_tank_ being already elevated, and therefore, attention shall be paid when the influence of different parameters to the *T*_tank_ is investigated. This phenomenon has also been demonstrated elsewhere (EPA 2014). From the available data, it seems that the influence of *T*_amb_ to the *T*_tank_ does not depend on the type or position of tank to the vehicle; however, more vehicles would need to be tested in order to reach a more definitive conclusion.

### Tank temperature during running operation

**Fig. 3:**
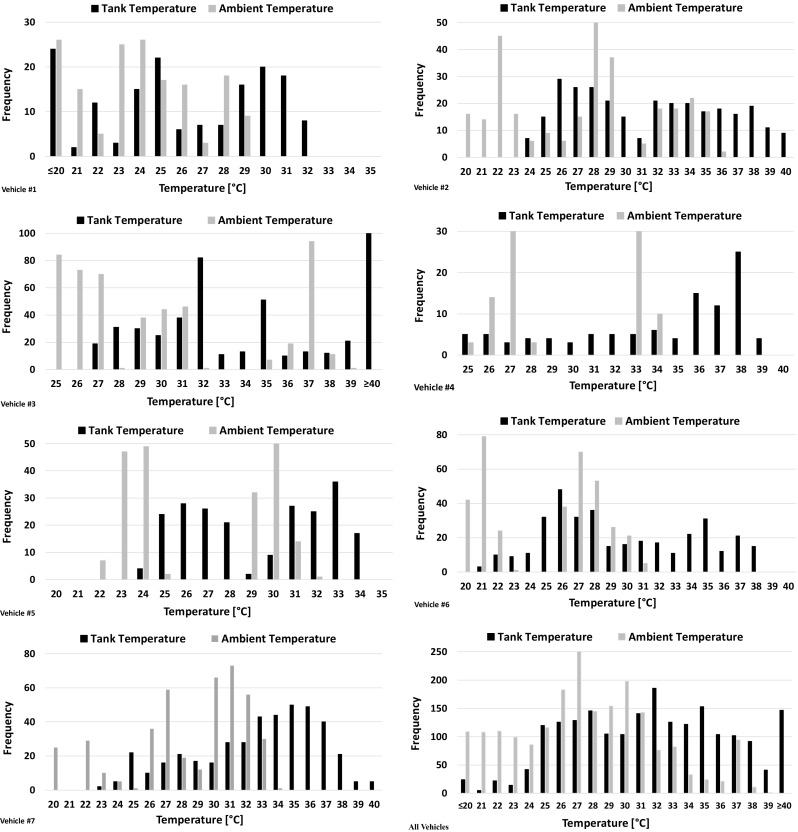
Distributions of the tank and ambient temperature recorded for all vehicles during running operation. The frequency refers to the number of recordings and corresponds to 1-min data for tank and ambient temperature

Figure 3 shows the *T*_tank_ and *T*_amb_ distributions recorded for all vehicles during running operation. *T*_tank_ was sampled with a frequency of 1 min; thus, the distributions for the individual vehicles were constructed based on 1-min *T*_tank_ and *T*_amb_ samples. In other words, the frequency in Fig. 3 corresponds to one 1 min for *T*_tank_ and *T*_amb_. For instance, a 5-min trip of a certain vehicle results in five different *T*_tank_ and *T*_amb_ values instead of a unique average value over the trip. For most vehicles, there is a wide distribution of *T*_tank_ between 25 and 40 °C, while *T*_amb_ ranged mostly between 20 and 35 °C. This is confirmed in Fig. 3 when the cumulative *T*_tank_ and *T*_amb_ distributions of all vehicles together are examined.

Tested vehicles can be separated into two categories based on the recorded *T*_tank_. The first category includes vehicles that demonstrate relatively low *T*_tank_. For instance, vehicle #1 shows *T*_tank_ distribution between 25 and 32 °C with no trips resulting in *T*_tank_ ≥ 35 °C. As seen in Fig. 3, the vast majority of the trips occur at relatively low *T*_amb_; therefore, *T*_tank_ is also low. On the other hand, some trips with vehicle #1 were done at higher *T*_amb_, but since they were of low/moderate speed and duration, they resulted in *T*_tank_ only up to 32 °C. Similarly, vehicle #5 demonstrates an equal distribution of *T*_tank_ between 25 and 34 °C without any trip coming with *T*_tank_ ≥ 35 °C. The first set of *T*_tank_ in the distribution (25–29 °C) presented in Fig. 3 reflects morning trips with relatively low *T*_amb_ (22–24 °C), while the second set (30–34 °C) reflects afternoon trips with higher *T*_amb_ (29–31 °C). However, *T*_tank_ does not reach higher values during afternoon trips due to low/moderate duration of individual trips and moderate vehicle speeds. For vehicle #6, *T*_tank_ is distributed between 25 and 38 °C. Approximately 20% of the running operation time results in *T*_tank_ ≥ 35 °C; however, it never exceeds 38 °C. In this case, trips #1 and #2 took place in the morning with *T*_amb_ of ~ 20 °C and resulted in *T*_tank_ of 25–29 °C. The rest of the trips took place over higher *T*_amb_ (26–30 °C) and resulted in a wide range of *T*_tank_ between 30 and 38 °C. Recorded high *T*_tank_ values of this vehicle during afternoon trips are linked to long duration of individual trips (> 25 min) and high vehicle speeds with some very high maximum speeds over the motorway phases. Overall, tests performed under low *T*_amb_ come with low/moderate *T*_tank_. Similarly, tests of short/medium duration and of low/medium vehicle speed performed under moderate *T*_amb_ come with moderate values for *T*_tank_ unless high trip duration and high vehicle speed is considered.

The second category includes vehicles that demonstrate generally high *T*_tank_ during running operation. This category is important for evaporative emissions as these emissions are clearly influenced by fuel temperature with any increase in its value resulting in significantly higher emissions compared with reference fuel temperature (Mellios et al. 2009). Vehicle #2 shows an approximately equal distribution of *T*_tank_ between 25 and 40 °C with one third of the testing time resulting in *T*_tank_ ≥ 35 °C. As explained in the trip analysis section, there are three kinds of trips for this vehicle: trips conducted in the morning with low *T*_amb_ resulting in *T*_tank_ of 25–27 °C, trips taking place under higher *T*_amb_ and resulting in *T*_tank_ > 30 °C, and follow-up (consecutive) trips which always result in *T*_tank_ > 33 °C. For vehicle #4, *T*_tank_ is distributed between 25 and 39 °C with 57% of the testing time coming with *T*_tank_ ≥ 35 °C. Three of the trips took place over low *T*_amb_ and resulted to *T*_tank_ of 25–30 °C. On the other hand, trips #1, #2, and #3 took place over higher *T*_amb_ (~ 33 °C) and resulted in *T*_tank_ up to 39 °C. High *T*_tank_ of trips #1 and #3 could also be linked to moderate/high duration and relatively high average speeds over these trips. Similarly, vehicle #7 showed a wide range of *T*_tank_ distribution with 40% of the testing time being with *T*_tank_ ≥ 35 °C. Trips for this vehicle took place either in the morning and came with low *T*_tank_ or in the afternoon and are linked to higher *T*_tank_. Trips of long duration (> 30 min) and high vehicle speed are also linked to high *T*_tank_. Finally, vehicle #3 was a different case as all trips took place in only 2 days. *T*_tank_ is distributed between 27 and 40 °C with half of the testing time coming with *T*_tank_ ≥ 35 °C. Early morning trips (#1 and #4) were done at *T*_amb_ of ~ 25 °C and resulted in the lower range of the *T*_tank_ distribution despite their high average speed and long duration, while the rest of the trips were conducted at higher *T*_amb_ and resulted in very high *T*_tank_. Overall, it can be concluded that high *T*_tank_ during running operation is a result of a combination of high *T*_amb_ or/and high vehicle speed and trip duration. Furthermore, it could be a result of multiple consecutive trips.

When all data are cumulated and plotted together (Fig. 3—all vehicles), a wide distribution of both *T*_tank_ and *T*_amb_ between 20 and 40 °C is revealed. Almost one third of the testing time is associated with *T*_tank_ ≥ 35 °C, while there is a 7% showing *T*_tank_ ≥ 40 °C. This range of temperature during running operation could prove crucial for evaporative emissions and raises questions about the actual *T*_tank_ over harder ambient temperature conditions usually met at the summer period in many Southern European countries.

### Difference between tank and ambient temperature during running operation

**Fig. 4:**
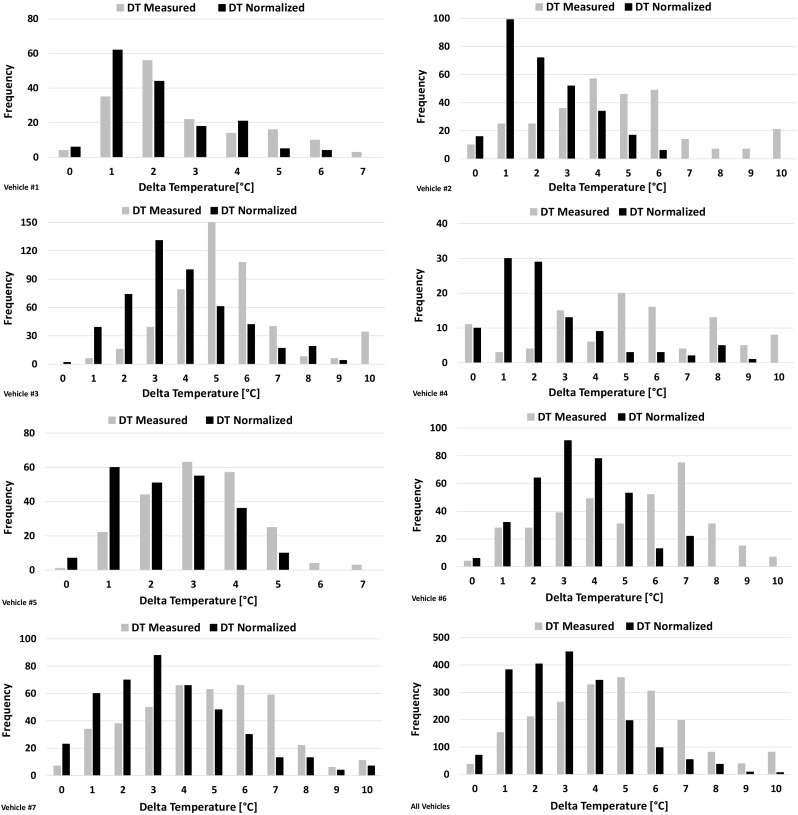
Distributions of the measured and normalized DT recorded for the individual vehicles and for all vehicles together during running operation. The frequency refers to the number of recordings and corresponds to 1-min data for both measured and normalized DT

This chapter provides information on how the DT (delta temperature = *T*_tank_ − *T*_amb_) evolves during running operation as well as how it is influenced by different parameters. As explained previously, there are several cases where two or more consecutive trips take place, and as a result, the initial *T*_tank_ of these trips is already elevated due to hot soak operation. In order to render all trips comparable with each other, it was decided to investigate the normalized DT which refers to the actual DT value corrected to the initial DT of each trip. For instance, if a trip starts with a DT of 3 °C, then all recorded values for this trip shall be reduced by 3 °C. In Fig. 4, both measured and normalized DT are plotted for all vehicles for reference, but the subsequent analysis is based solely on the normalized values. Like in the case of Fig. 3, the *Y*-axis frequency corresponds to 1-min data recordings for both measured and normalized DT values. Thus, an *X*-minute trip results in *X* different DT values instead of a unique average DT value over the trip.

Figure 4 shows that for all vehicles, DT is distributed between 1 and 10 °C. In most cases, normalized DT lies within the range of 1–5 °C, while the 50th percentile of the distribution does not exceed 3 °C. Since borderline conditions are more important for evaporative emissions, the 95th percentile of the DT distributions was also examined and found to vary between 4 and 8 °C depending on the testing conditions. For vehicle #1, normalized DT did not exceed 6 °C with the 50th and the 95th percentiles of the distribution being at 1.3 °C and 4.2 °C, respectively. Short–medium duration and moderate vehicle speed of the trips—along with relatively low *T*_amb_ during testing—explain the generally low DT values. The same applies to vehicle #2 where the 50th and the 95th percentiles of the distribution were found at 1.4 °C and 4.5 °C, respectively. Despite that vehicle #2 was tested over generally higher *T*_amb_ compared with vehicle #1, no significant differences in the DT were found, thus showing that *T*_amb_ is not so crucial for DT when short/medium trips of low/moderate speed are considered. Similarly, normalized DT values of vehicle #5 did not exceed 5 °C with the 50th and the 95th percentile of the distribution being at 1.9 °C and 4.0 °C, respectively. Vehicle #4 was tested over similar conditions but at slightly higher *T*_amb_. Despite that DT varied more and reached even 9 °C in some trips, the 50th percentile of the distribution was found to be at 1.5 °C. However, the 95th percentile was found to be significantly higher reaching 7.1 °C and reflecting the difference in the *T*_amb_ during testing. Overall, it can be concluded that vehicles driven over short/moderate trips with moderate average speed usually exhibits low to moderate DT values. DT values higher than 5 °C may occur under these circumstances, but the frequency of occurrence is very low compared with lower DT values.

Vehicles #3, #6, and #7 exhibited slightly different DT distributions compared with the ones described previously for the rest of the vehicles. For vehicle #3, DT turned out to cover a wide range of temperatures (1–10 °C) with the 50th and the 95th percentiles of the distribution being at 3 °C and 7 °C, respectively. The same applies to vehicle #7, while vehicle #6 showed a DT distribution within the range of 1–7 °C with the 50th percentile being at 2.8 °C and the 95th percentile at 6.2 °C. Higher average vehicle speeds and longer duration of the trips explain the shift of the distribution towards higher DT values for these vehicles. When cumulative data from all vehicles are examined (Fig. 4—all vehicles), approximately 10% of the running operation time comes with a normalized DT ≥ 5 °C, while the 50th and the 95th percentiles of the distribution lie at 2.5 °C and 6.1 °C, respectively.

#### Influence of trip duration on the DT

**Fig. 5:**
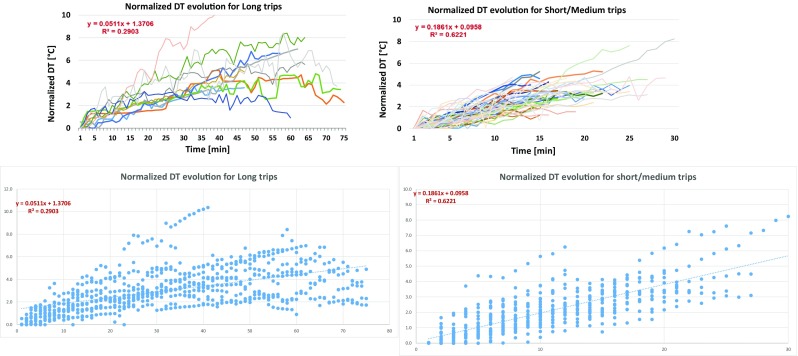
Summarized minute-to-minute evolution of normalized DT for trips ≤ 30 min and trips > 30 min

Figure 5 demonstrates the minute-to-minute evolution of normalized DT values for all trips. For better illustration purposes, Fig. 5 is divided into two parts. Figure 5a depicts all trips with a maximum duration of 30 min and Fig. 5b all trips with a duration exceeding 30 min.

Figure 5a shows that for short/medium duration trips, there seems to be a satisfactory correlation between the normalized DT and the trip duration (*R*^2^ = 0.6221). All trips show a DT increase of 2–4 °C for the first 15 min of the trip, while later on, DT increases up to 5–8 °C depending on other parameters like the vehicle speed. An equation supposing normalized DT as a function of traveling time is also provided in Fig. 5; however, one should keep in mind the limitations when applying this function (i.e., different vehicles, different speed profiles, etc.). The picture appears to be quite different when long trips (> 30 min) are examined. In Fig. 5b, it is demonstrated that while for the initial 30 min there seems to be a similar behavior as for short/medium trips, after 30 min, some trips exhibit further increase of the DT while others show a stabilization (or even a slight decrease) of normalized DT with the time. This is very well reflected by the significantly reduced correlation between the normalized DT and the trip duration (*R*^2^ = 0.2903). This phenomenon is not vehicle specific but was observed in all vehicles tested over long trips. It can be partly attributed to the fact that DT depends not only on the trip duration but also on vehicle speed. Tests showing reduction of DT after 30 min could be linked to vehicles following a moderate speed with medium engine load, while tests with increasing DT can be linked to extremely high speeds in the motorway. Finally, in some cases, it is possible that DT reaches the maximum possible value depending on the maximum temperature that the fuel can reach in the tank; therefore, DT appears in Fig. 5 as a saturated curve. Unfortunately, not many trips of very long duration where performed in order to have enough data to statistically analyze the difference and reach a solid conclusion.

**Fig. 6:**
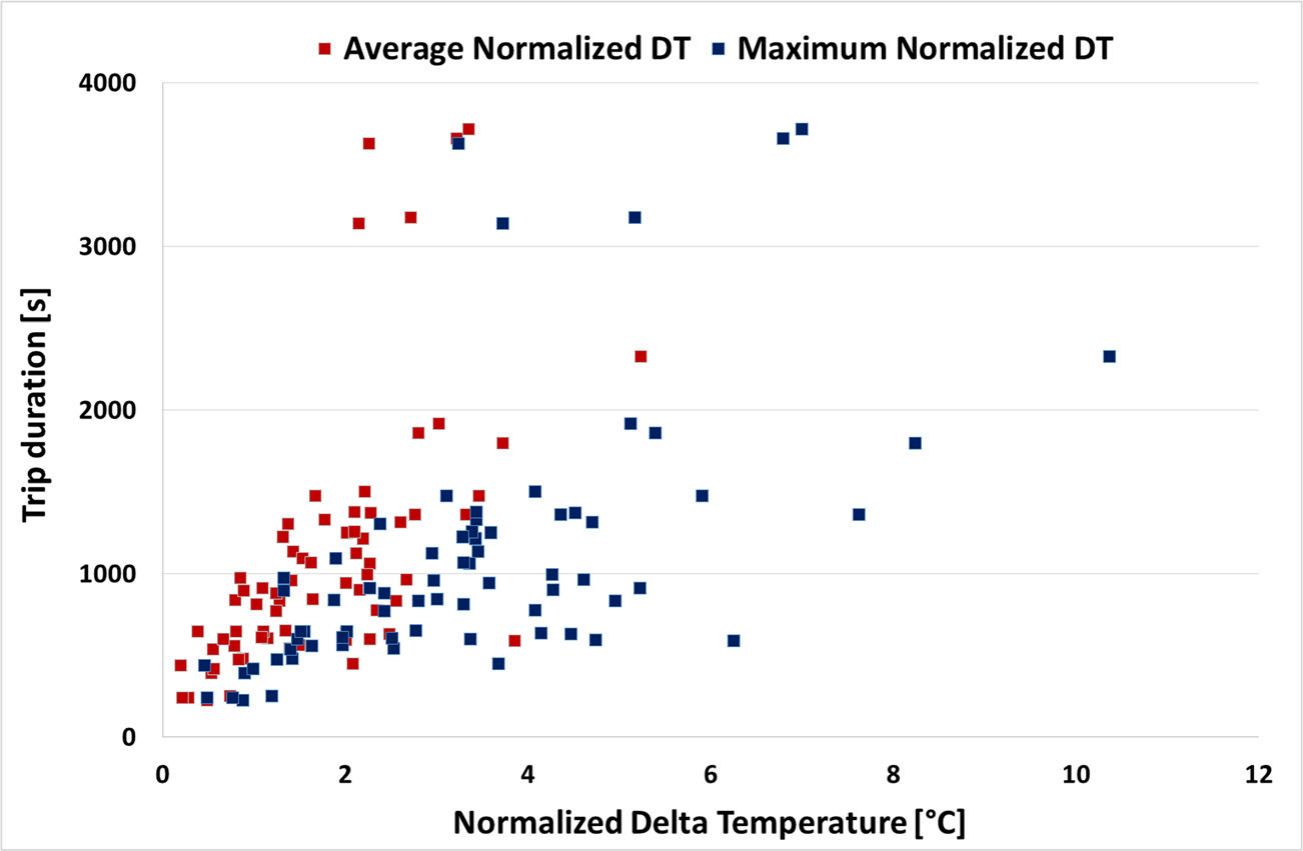
Average and maximum value of the normalized DT for each trip plotted against the trip duration for all trips

Figure 6 depicts the average and maximum value of the normalized DT for each trip plotted against the duration of the trip. Figure 6 confirms the trends described previously. It seems that for most vehicles, there is a tendency for increased average DT with trip duration up to a certain level. The effect becomes more pronounced when the maximum DT of the individual trips is examined. Short trips (< 10 min) rarely exceed average DT of 3 °C, while very long trips usually reach high maximum DT values (> 5 °C) but not very high average DT values suggesting that there is a stabilization of the normalized DT after a certain point (i.e., 30 min). Table 3 also confirms these findings as it is suggested that increased trip duration leads to increased average and maximum DT.

**Table 3:** Average and maximum normalized DT for each trip category as defined in Table 2

Trip category	Average normalized DT (°C)	Maximum normalized DT (°C)
Short duration	1.1	2.0
Medium duration	1.8	3.5
Long duration	3.3	5.8
Low speed	1.2	2.2
Medium speed	2.0	3.7
High speed	3.4	5.6

#### Influence of vehicle speed on the DT

**Fig. 7:**
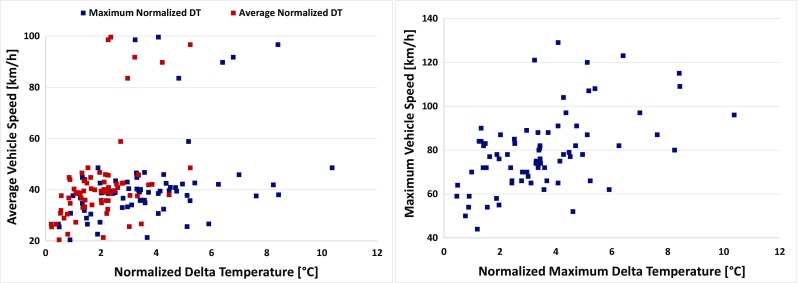
Average and maximum value of the normalized DT for each trip plotted against the average vehicle speed for all trips and normalized maximum DT value for all trips plotted against the maximum vehicle speed

Figure 7a depicts the average and maximum normalized DT values for each one of the total of 78 trips plotted against the average vehicle speed. It seems that for most trips, the average DT does not strongly correlate with the average vehicle speed (*R*^2^ = 0.2404). Trips with low average speed (< 30 km/h) always come with lower average and max DT (< 3 °C), but trips with higher average speed (> 70 km/h) come with a wide range of DT—both average and maximum—pointing thus to DT dependency on other parameters rather than the average speed. It should be noted that Table 3 indicates a dependency of average and maximum DT to the vehicle speed; however, the small sample of high speed trips (*n* = 6) does not allow for statistical confirmation of this correlation. Maximum normalized DT was also examined, but it seems that it does not correlate better to the average speed (*R*^2^ = 0.173) than the average normalized DT (*R*^2^ = 0.2404). This means that the average speed of a trip does not significantly affect neither average nor maximum normalized DT values.

Figure 7b plots the maximum value of the normalized DT for each trip against the maximum vehicle speed during the trip. No good correlation between these values was observed (*R*^2^ = 0.226). Urban trips with low maximum speed (< 50 km/h) tend to come with lower maximum DT values (< 2 °C); rural trips (< 80–90 km/h) mostly come with a wide range of maximum DT values but never exceed 6 °C, while motorway trips (> 90 km/h) occur over maximum DT values which range from 4 to 10 °C. Overall, it seems that the maximum vehicle speed does not provide more information than the average vehicle speed when trying to predict the DT behavior of a vehicle.

#### Influence of tank level on the DT

**Fig. 8:**
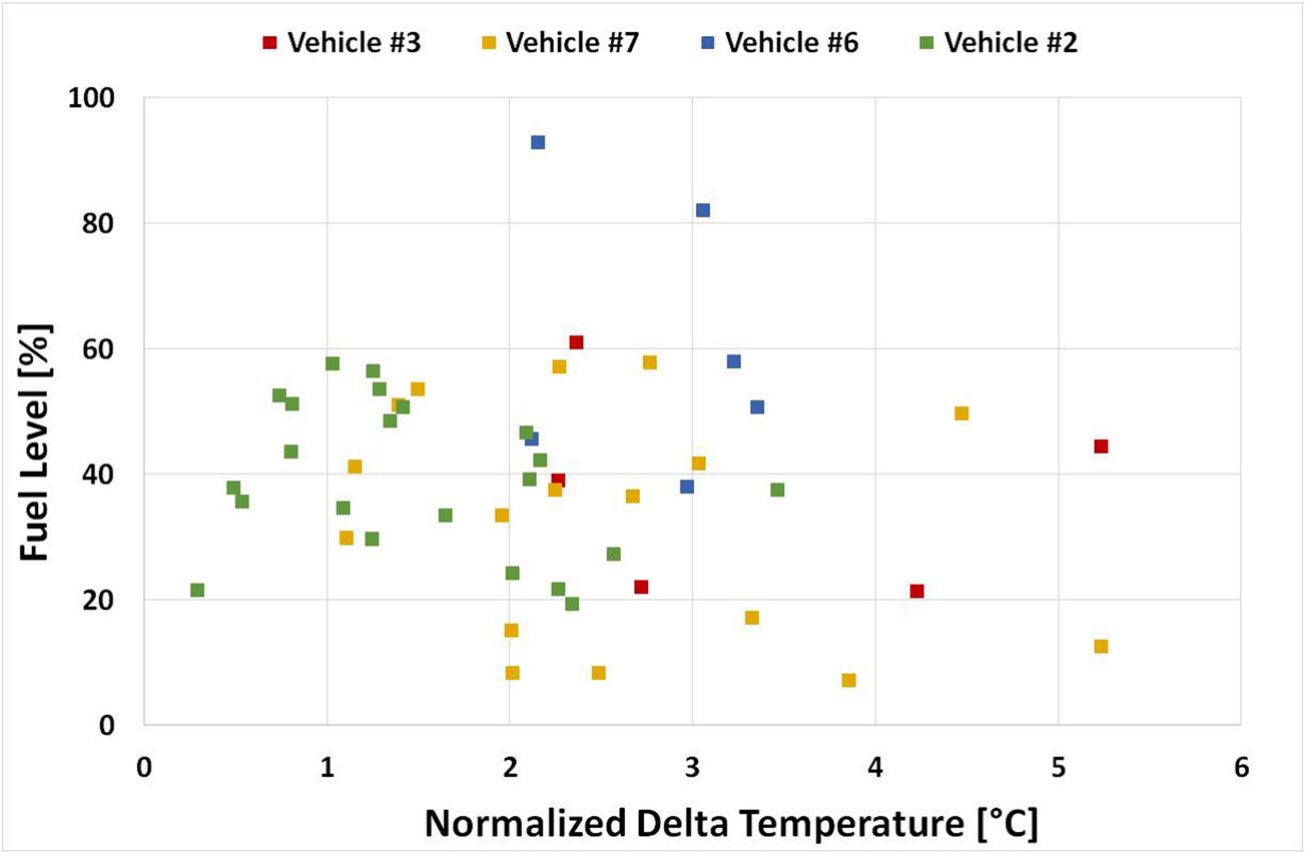
Average normalized DT for each trip plotted against the average fuel level during running operation for vehicles #2, #3, #6, and #7

For the most recently manufactured vehicles (#2, #3, #6, #7), fuel level data during running operation were recorded (OBD). Figure 8 depicts the average normalized DT for each trip plotted against the average fuel level during the trip. The aim was to understand the influence of the tank level on the average DT. Figure 8 shows that trips with relatively low fuel tank level (< 20%) come with a wide spread of average DT values (2–6 °C). The same applies for trips with medium and high fuel tank level, thus not allowing for any dependence among the examined parameters to be established. This seems to be the case for all examined vehicles. However, vehicles #3 and #7 showed generally higher DT values compared with vehicles #2 and #6. This may relate to the dimensions of the fuel tank which for these vehicles is close to 50 L (Table 1) whereas for vehicles #2 and #6 is 40 L. In any case, more data is required to establish a correlation between the tank dimensions and the DT.

### Purging of the canister

**Fig. 9:**
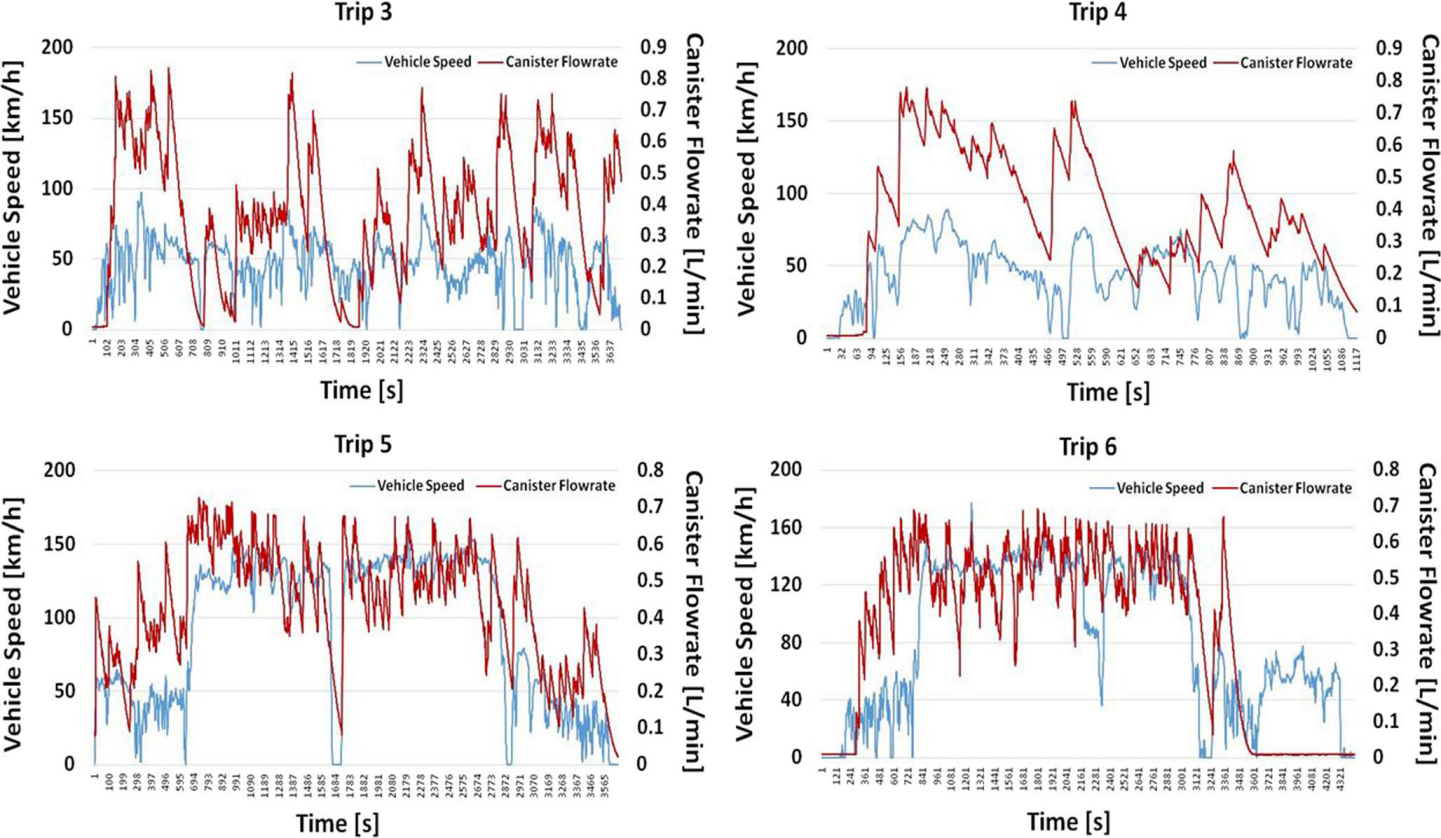
Second-by-second canister flow rate vs. speed trace of four individual trips for vehicle #6

Figure 9 presents the recorded canister flow rate as well as the speed trace of four individual trips for vehicle #6. Some statistics of these trips are also provided in Table 4. It is reminded that the flow meter is unidirectional meaning that it does not provide information whether gas vapors are flowing outside the tank or ambient air is sucked in it. It can be seen from Fig. 9 that the canister flow rate follows the general speed trend. For cold start trips, the flow rate initially seems to be at a quasi-zero level, but after approximately 5 min, it increases to levels varying between 0.1 and 0.7 L/min. This observation was also confirmed from laboratory tests which are not presented here. Hot start tests—which in our case represent consecutive trips—did not demonstrate this phenomenon (trip 5). During trip #6, the driver switched the vehicle from conventional to electric mode for the final 15 min of the test and the canister stopped purging as it can be seen in Fig. 9d.

**Table 4:** Average statistics from vehicle #6 (four individual trips) and vehicle #7 (three individual trips)

	Vehicle #6				Vehicle #7		
	Trip 3	Trip 4	Trip 5	Trip 6	Trip 3	Trip 11	Trip 16
Canister flow rate (L/min)	0.38	0.39	0.43	0.36	1.30	0.82	0.89
Fuel level (%)	50.8	45.7	58.0	38.1	12.7	49.7	33.4
*T*_amb_ (°C)	25.9	29.9	27.0	28.4	28.5	30.0	28.3
Vehicle speed (km/h)	45.9	43.0	91.8	83.5	48.6	44.4	46.8
Total volume (L)	23.25	7.28	26.43	26.58	50.53	49.87	62.98

Table 4 shows that there is no clear correlation between the average canister flow rate with neither of the parameters examined. Trips #3 and #4 were conducted with similar medium average speed and gave similar canister flow rates to trips #5 and #6 which were performed over double as high average speeds. *T*_amb_ did not differentiate significantly among the trips; however, when trips #3 and #4 are compared with each other, they practically demonstrate the same canister flow rate despite the 4 °C difference in the *T*_amb_. Similarly, Mellios et al. (2009) reported similar canister flow rates for the EUDC and UDC cycles with a VW Polo tested with different fuels and *T*_amb_. Finally, regarding the fuel level, no safe conclusions can be drawn since all trips were done with similar fuel tank level. More tests—preferably with a bi-directional flow meter—are required to better understand the purging strategy of vehicle #6.

**Fig. 10:**
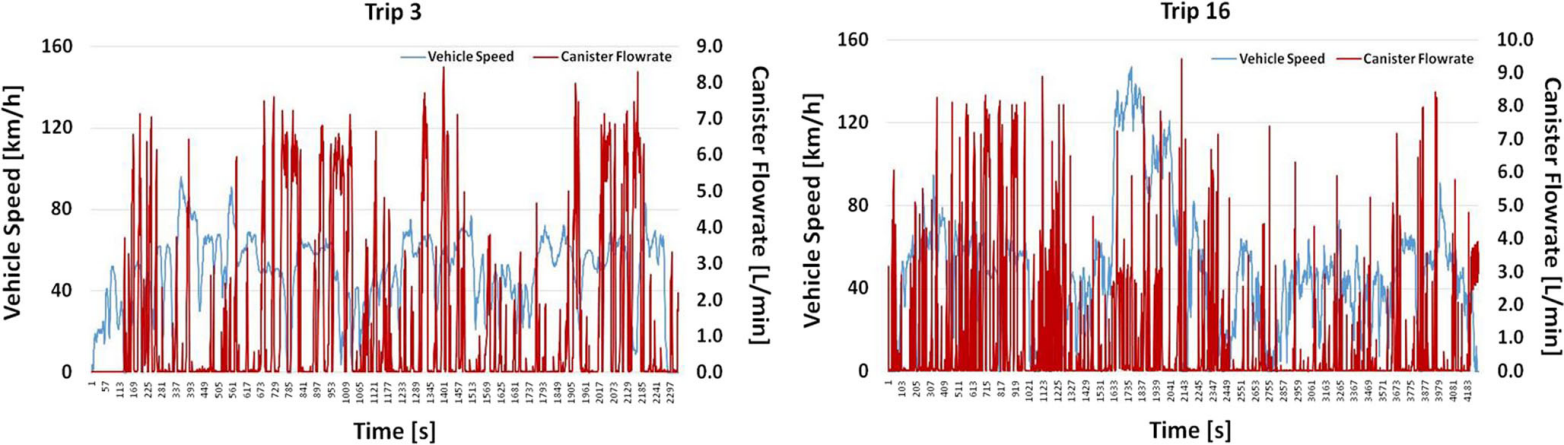
Second-by-second canister flow rate vs. speed trace of two individual trips for vehicle #7

A similar exercise was also performed for some trips of vehicle #7 with the aim of understanding if different vehicles demonstrate similar purging strategies. Figure 10 presents the recorded canister flow rate as well as the speed trace of two individual trips for vehicle #7. Some statistics of these trips (plus an additional trip) are also provided in Table 4.

It can be seen from Fig. 10 that the canister flow rate does not follow the vehicle speed trend as there are frequent flow rate peaks not linked to the speed trace. Table 4 confirms that there is no correlation between the average canister flow rate with the examined parameters. Actually, trips conducted with similar *T*_amb_ and vehicle speed result in different canister flow rate levels. It could be the case that *T*_amb_ is one of the parameters used by the manufacturer to define the purging strategy; however, this is not known and is also not proven by available data. Regarding the fuel level, it seems that low fuel levels can result in higher flow rate in accordance with what has been reported in the literature (Yamada 2013), but this is observed only in one trip, and thus, no safe conclusion can be drawn. Overall, three to four times higher canister flow rate levels compared with vehicle #6 are observed pointing to different purging strategies. Since the parameters used by the manufacturers to define the individual purging strategies are not known, no safe conclusion can be drawn. More tests—preferably with a bi-directional flow meter—under different conditions are required to better study and understand the purging strategy of both vehicles.

## Conclusions

The main objective of this study was to investigate the *T*_tank_ behavior of different vehicles tested under different operating conditions. The main outcomes of the analysis can be summarized to

- *T*_tank_ mainly depends upon the *T*_amb_ following its trend. This is quite important as high *T*_amb_ (≥ 35 °C) is frequent during the summer period specifically in Southern Europe and may result in high evaporative emissions to the atmosphere under specific circumstances.
- Trip duration and driving pattern also influences *T*_tank_. *T*_tank_ differentiates from *T*_amb_ (increases) during running operation and particularly when long trips combined with high vehicle speed are examined. The same effect is observed when multiple consecutive trips take place. When such trips take place under relatively hot conditions, it is often the case that *T*_tank_ exceeds 35 °C (30% of the trips) and reaches even 40 °C (5% of the trips).
- Combined higher average vehicle speed and longer trip duration result in higher DT (delta temperature = *T*_tank_ − *T*_amb_) values compared with more moderate conditions. *T*_amb_ does not seem to have a significant effect on DT. Normalized DT values higher than 5 °C were observed, however with much lower frequency compared with lower DT values.
- There is a tendency of increase of the average DT value of the trip with the duration. This is observed for the first 30 min, while afterwards, DT seems to stabilize or in some cases fluctuate. On the other hand, in most cases, the average DT value does not strongly correlate to the average vehicle speed; however, the small sample of high average speed trips does not allow for a solid conclusion to be drawn. Finally, no correlation between the tank fuel level during running operation and the DT was established.
- Two of the examined vehicles demonstrated completely different purging strategies to each other. Vehicle #7 exhibited three to four times higher purging flow rate levels compared with vehicle #6. Purging flow rate does not seem to correlate with none of the examined parameters; however, more tests preferably with a bi-directional flow meter are required to investigate potential running losses.

Based on the findings of the current study, it became obvious that further investigation with special focus to the influence of “extreme” environmental conditions (i.e., *T*_amb_ > 35 °C) on parameters such as the *T*_tank_ and the purging strategy is required. For that reason, a series of experiments under controlled environmental conditions in the laboratory have been scheduled for investigating running losses of Euro 6 vehicles with the use of a bi-directional flow meter.

## Electronic supplementary material

ESM 1 (XLSX 569 kb)

ESM 2 (XLSX 759 kb)

ESM 3 (XLSX 1247 kb)

ESM 4 (XLSX 300 kb)

ESM 5 (XLSX 530 kb)

ESM 6 (XLSX 1252 kb)

ESM 7 (XLSX 1295 kb)
